# Temporal and Regional Regulation of Gene Expression by Calcium-Stimulated Adenylyl Cyclase Activity during Fear Memory

**DOI:** 10.1371/journal.pone.0013385

**Published:** 2010-10-14

**Authors:** Lindsay Wieczorek, James W. Maas, Lisa M. Muglia, Sherri K. Vogt, Louis J. Muglia

**Affiliations:** 1 Departments of Pediatrics and Developmental Biology, Washington University, Saint Louis, Missouri, United States of America; 2 Departments of Pediatrics and Molecular Physiology and Biophysics, and Vanderbilt Kennedy Center, Vanderbilt University, Nashville, Tennessee, United States of America; Yale School of Medicine, United States of America

## Abstract

**Background:**

The Ca2+-stimulated adenylyl cyclases (ACs), AC1 and AC8, are key components of long-term memory processing. AC1 and AC8 double knockout mice (Adcy1^−/−^Adcy8^−/−^; DKO) display impaired fear memory processing; the mechanism of this impairment is largely unknown.

**Methodology/Principal Findings:**

We hypothesize that the Ca2+-stimulated ACs modulate long-lasting transcriptional changes essential for fear memory consolidation and maintenance. Here, we report a genome-wide study of gene expression changes associated with conditioned fear (CF) memory in wild-type and DKO mice to identify AC-dependent gene regulatory changes that occur in the amygdala and hippocampus at baseline and different time points after CF learning. We observed an overall decrease in transcriptional changes in DKO mice across all time points, but most strikingly, at periods when memory consolidation and retention should be occurring. Further, we identified a shared set of transcription factor binding sites in genes upregulated in wild-type mice that were associated with downregulated genes in DKO mice. To prove the temporal and regional importance of AC activity on different stages of memory processing, the tetracycline-off system was used to produce mice with forebrain-specific inducible expression of AC8 on a DKO background. CF behavioral results reveal that adult restoration of AC8 activity in the forebrain is sufficient for intact learning, while cessation of this expression at any time point across learning causes memory deficits.

**Conclusions/Significance:**

Overall, these studies demonstrate that the Ca2+-stimulated ACs contribute to the formation and maintenance of fear memory by a network of long-term transcriptional changes.

## Introduction

The cAMP signal transduction pathway has been repeatedly implicated in learning and memory using both invertebrate and vertebrate models. More specifically, the Ca2+-stimulated adenylyl cyclase (AC) pathway, which couples neuronal activity and intracellular Ca2+ increases to the production of cAMP, is crucial for normal memory processes [1]. This essential role is evident by memory impairments seen in the rutabaga *Drosophila* mutant, which shows a lack of Ca2+-stimulated AC activity [2]. Of the ten AC isoforms in mammals, AC1 and AC8 are the only two that are primarily stimulated by Ca2+/calmodulin [3], [4], [5]. Murine models have demonstrated the importance of these isoforms in memory processing. For example, both AC1 knockout (AC1KO) and AC8 knockout (AC8KO) mice display learning impairments in the Morris water maze [6], [7]. Moreover, there appears to be functional redundancy in these two isoforms as passive avoidance and conditioned fear (CF) memory are intact in AC1KO or AC8KO mice but are impaired in AC1 and AC8 double knockout (DKO) mice [5]. Interestingly, DKO mice show normal CF memory at 24 hr, but not 1 wk, suggesting that Ca2+-stimulated activity is necessary for long-term memory changes.

AC1 and AC8 are both localized to brain regions known to play essential roles in memory processing, such as the cortex, cerebellum, and hippocampus [8], [9], [10]. At the cellular level, AC1 and AC8 are localized to the synapse, specifically the postsynaptic region for AC1 and presynaptic region for AC8 [11]. The regional and subcellular location of these two isoforms clearly has physiological implications as AC1KO and AC8KO mice show impairments in mossy fiber long-term potentiation (LTP) [12].

Although Ca2+-stimulated AC activity has been implicated in modulating behavior, the mechanism by which this occurs has still not been thoroughly defined. There is evidence highlighting deficits in acute, short-term activation of the MAPK/ERK pathway 30 min after CF training [13]. However, since long-term memory and LTP are both dependent on transcription and are disrupted in AC knockout models, we hypothesize that the primary effect of Ca2+-stimulated AC activity during CF is to modulate gene expression [14], [15], [16] We assessed the effect of Ca2+-stimulated AC activity on global gene expression via microarray analysis. The contextual CF paradigm, which relies on the structural integrity of the hippocampus and amygdala [17], was used as our paradigm to define the network changes that result during memory processing in the context of disruption and rescue of AC expression with knockout and transgenic mouse models. We demonstrate that Ca2+-stimulated AC activity is necessary during memory consolidation and retention and that there is an overall attenuation of transcriptional changes in mice lacking both Ca2+-stimulated AC isoforms.

## Materials and Methods

### Animals

All mouse protocols were in accordance with National Institutes of Health guidelines and were approved by the Animal Care and Use Committees of Washington University School of Medicine (St. Louis, MO) (protocol approval #20080030) and Vanderbilt University (Nashville, TN) (protocol approval #M08617). Mice were housed on a 12 hr/12 hr light/dark cycle with *ad libitum* access to rodent chow and water. For control of the inducible tetracycline-off system, mice were either fed doxycycline chow (200 mg doxycycline/1 kg; Research Diets) to repress transgene expression or fed normal rodent chow to permit transgene expression.

DKO [5], [6], AC1KO [6] and AC8KO [18] mice were generated as previously described. To produce forebrain-specific, inducible AC8 expression mice (AC8 rescue) on a DKO background, a tetracycline-off system was used to allow for temporal control over AC8 cDNA expression. The tetracycline-off system is based on the interaction of a tetracycline transactivator (tTA) with a tetracycline-responsive element (tetop) [19], [20], [21]. In the presence of tetracycline or doxycycline, tTA loses its ability to bind tetop and expression is turned off. In our system, we have inserted AC8 cDNA under the control of a tetracycline-responsive CMV minimal promoter, generating tetop-AC8 mice. The linearized sequence was microinjected in C57Bl/6 oocytes and founder lines were identified. To confirm that the founder lines were capable of inducible AC8 cDNA expression, they were mated with tTAluc mice [22], which express tTA in many tissues with no detectable endogenous AC8 expression, allowing us to establish that the transgene was expressed (data not shown). Once inducible expression of the AC8 cDNA was established, mice were mated with CaMKII-tTA mice (CaMKII-tTA mice from Jackson Laboratories) [23], which have tTA under control of the CaMKII forebrain-specific promoter. CaMKII-tTA and tetop-AC8 mice were then mated to DKO mice to generate AC8 rescue mice. Each separate line was maintained on an inbred C57BL/6 background. All AC8 rescue matings were on doxycycline and pups were kept on doxycycline until weaned at 21 days to keep AC8 off during development, which allows us to truly assess the learning changes that result from acutely replacing Ca2+-stimulated AC activity in the forebrain of developed DKO mice. DKO mice used in the present studies were mice positive for the tetop-AC8 transgene or CaMKII-tTA transgene alone or wild-type littermates of the AC8 rescue mice. C57Bl/6 were used as non-littermate control mice (WT).

### Tissue collection and RNA extraction

We collected hippocampal and amygdala samples from WT and DKO mice at baseline and four different time points after a 5 min CF training trial. The four time points (and the respective memory stages each time point corresponded to) were as follows: 0 hr (acquisition), 1 hr (consolidation), 48 hr (retention), and 1 wk after CF training (retrieval). For the 1 wk after CF training time point, mice were tested in a 5 min CF testing trial prior to killing. Micropunches using a 1 mm diameter capillary tube were used to extract bilateral hippocampal and amygdala punches approximately 2 mm thick. Tissue was quickly preserved using RNA later (Qiagen) at 4°C and moved to −80°C 24 hr later until RNA preparation. The Qiagen miRNAeasy Mini Kit was used to extract RNA, which uses a two-step process, both Trizol and column extraction, to isolate purified, intact RNA.

### Microarray analysis

RNA samples from 5–10 mice were pooled per array with one array per genotype/brain region/time point for a total of 20 arrays. Prior to processing the tissue for microarray analysis, RNA quality was verified for each sample by an Agilent 2100 Bioanalyzer and only samples with a RIN value greater than 8.0 were used. mRNA was then reverse transcribed, labeled, and hybridized to Affymetrix Mouse Gene ST 1.0 Arrays (∼29,000 transcripts) by the Vanderbilt Functional Genomics shared resource core using standard procedures. The Affymetrix Gene Chip Command Console was used for all instrument control and data acquisition, while the Expression Console (Affymetrix) was used for normalization (RMA) and primary data analysis. All microarray data is MIAME compliant and the raw data has been deposited in Gene Expression Omnibus (http://www.ncbi.nlm.nih.gov/geo/) (GEO accession number: GSE23008), while fold change (fc) values relative to baseline can be found in Tables S1, S2, S3, S4. Genes that were further characterized and thought to be of interest were categorized into two lists. One list represents genes that did not change over time despite exposure to CF testing, but remained consistently different between genotypes (Table 1). These genes were classified as developmental gene changes that resulted from knocking out AC1 and AC8 from birth. Statistical analysis here pooled across the time points and considered each time point to an n of 1 per genotype (total of 5 arrays/genotype/brain region). We used Qvalue [24] to assess statistically significant gene changes across the arrays comparing DKO and WT mice, which takes a list of p-values resulting from the simultaneous testing of many hypotheses and estimates their q-values in order to determine the false discovery rate. A q-value of ≤0.1 was considered significant. The other list represents genes that changed acutely as a result of learning changes and these genes displayed at least a ±1.5 fc relative to baseline at one or more time points (Table S5 and S6). These genes could not be assessed statistically as only one array was run per condition.

**Table 1:** Genes that are modulated during development.

Amygdala			
Accession ID	Gene symbol	Mean fold change	P-value
NM_008732	Slc11a2	−1.32	2.8E-08
NM_009623	Adcy8	−2.76	3.8E-07
NM_025931	Rabl4	−1.38	9.9E-07
NM_146014	Ccm2	−1.37	1.2E-05
NM_146776	Olfr821	1.17	2.9E-05
NM_029787	Cyb5r3	−1.64	3.9E-05

Hippocampus			
Accession ID	Gene symbol	Mean fold change	P-value
NM_009622	Adcy1	−2.97	6.2E-08
NM_009623	Adcy8	−4.06	4.8E-07
NM_011838	Lynx1	−1.40	2.1E-06
NM_011124	Ccl21a	−1.64	3.1E-06
NM_023052	Ccl21c	−1.64	3.1E-06
NM_011335	Ccl21b	−1.64	3.1E-06
NM_175408	Tmem139	−1.22	1.9E-05
NM_009342	Dynlt1	1.26	6.0E-05
NM_029787	Cyb5r3	−1.47	7.3E-05
NM_026681	Ccdc88c	−1.18	7.7E-05

The genes, with their respective fold change (from WT level) and p-value, are listed in order of significance. Only genes that met a q-value of ≤0.1 were listed.

### Real time RT-PCR

The same individual RNA samples used on the microarrays were also used for the microarray RT-PCR validation experiments. To prepare the cDNA, 500 ng of total RNA was reversed transcribed in a 20 uL reaction using the High Capacity cDNA Reverse Transcription Kit (Applied Biosystems). Real time RT-PCR was performed with SYBR Green Master Mix (Sigma Aldrich) in an Applied Biosystems 7900HT Fast Real-time PCR System at Vanderbilt University's DNA Resource Core. Each sample was run in duplicate and dissociation curves were used to assess the specificity of each primer pair. GAPDH was used as the standard control. Primers used for amplification are listed in Table S7. For RT-PCR validation of microarray results, a random selection of genes was evaluated in WT and DKO mice across all time points in the amygdala and hippocampus to confirm that microarray results correlated with RT-PCR results across all arrays (Fig. S1).

### Functional analysis

To assess the most represented functional annotations among the genes that showed a ±1.5 fc relative to baseline, the GO Slimmer program from Gene Ontology (www.geneontology.org) was used. The number of genes falling into each functional annotation was determined to assess the most represented functional annotations per condition. A minimum parameter of 3 was used. Any GO category whose level in the GO hierarchy is below this parameter was not included in the GO analysis in order to eliminate functional categories that are too broad. The category, Biological Process, is defined to be at level 1 in the hierarchy. The level of any other term is the length of the longest path to level 1 in terms of the number of categories on the path.

### Gene cluster analysis

The software program Short Time-Series Expression Miner (STEM) [25] was used to cluster and analyze the gene expression data from the microarray experiments. Genes were filtered so that only genes with a ±1.5 fc at one or more time points were assigned to a cluster. The STEM program calculates the significance of a cluster based on the ratio of the number of assigned genes versus the number of expected genes to a profile. The program has the ability to implement a novel method for clustering short time series expression data that can differentiate between real and random patterns as previously described [26].

### Transcription factor analysis

We used Whole Genome rVISTA (http://genome.lbl.gov/vista) to identify transcription factor binding sites that are conserved between mice and humans and overrepresented in the 2.5 Kbp upstream region of genes that were regulated similarly during the CF paradigm.

### Immunohistochemistry

Tissue slices were stained for AC8 as described previously [11]. Briefly, mice were anesthetized and transcardially perfused with 4% paraformaldehyde. Frozen tissue was cut at 30 µm and sections were incubated in goat anti-AC8 antibody (1∶400, Santa Cruz Biotechnology) overnight at 4°C. Sections were then incubated with a biotinylated rabbit anti-goat secondary antibody (Vector Labs) at 1∶800 for 1 hr. Biotin was detected with diaminobenzidine and sections were slide mounted with DAPI, a nuclear stain. All images were obtained using matched settings between genotypes on an Olympus BX60 fluorescent microscope equipped with Axiovision software. Images were prepared using Adobe Photoshop.

### Western blot analysis

AC8 protein levels were assessed in mice at various time points after doxycycline treatment as described previously [11]. Briefly, mice were killed by CO_2_ inhalation and the brains rapidly removed. Subregions were dissected on ice, snap frozen in liquid nitrogen and stored at −80°C. Frozen tissues were homogenized with a lysis buffer containing a protease inhibitor and phosphatase cocktail and protein concentration was determined by the BCA assay (Pierce Biotechnology). For AC8 detection, 20 µg of membrane extract from each region was separated by 4–12% SDS-PAGE, transferred to nitrocellulose membrane and immunoblotted with goat anti-AC8 antibody (1∶500, Santa Cruz Biotechnology) overnight at 4°C. Equal protein loading conditions were verified by immunodetection for mouse anti-binding protein (BiP) in all samples. Signals were detected using an anti-goat HRP-conjugated secondary antibody, and visualized using chemiluminescence (SuperSignal WestDura; Pierce Biotechnology).

### Adenylyl cyclase assay

Brain regions were excised from WT, DKO, and AC8 rescue mice and assayed for AC activity as previously described [27]. Free Ca2+ concentrations were calculated using the Bound and Determined computer algorithm [28]. AC activity levels are the means of triplicate samples. Protein concentration in the cell membranes was determined as previously described [29].

### Behavioral analysis

An observer blinded to genotype performed all behavioral analysis. Behavioral experiments were conducted 2–5 hr after lights on. Male mice ages 2–4 mo were used for all behavioral experiments, except for the first CF experiment, where male and female mice ages 2–8 mo were used. All mice were on a C57Bl/6 inbred background.

#### Conditioned Fear

Cognitive skills were evaluated using a test of Pavlovian fear conditioning. The CF chamber was a standard grid box (20.3×15.9×10.0 cm; Med Associates) with a small vial of coconut oil, which served as an olfactory cue. During CF training, mice were put in the chamber for 2 min to assess basal (pretraining) freezing levels, followed by 28 sec of white noise and a 2 sec 0.7 mA foot shock (white noise was used for cued CF but data is not shown). Subsequently, postshock (posttraining) exploration was monitored for an additional 2 min. Freezing was monitored in 5 sec bins. Graphs express the percent freezing over the course of a testing period. 1 wk, 1 mo and 1.5 mo after training, mice were put back in the original training chamber to assess contextual CF memory. Freezing was monitored as before for 5 min. Doxycycline was given or removed for at least 2 wk, in most cases a month, in between testing to allow for AC8 expression to be turned on or off effectively.

#### Novel object recognition

The mice were habituated to the testing chamber for 4 hr before training. Following habituation, two different shaped blocks were presented during a 10 min training session. Testing occurred 90 min later with mice being presented with one familiar object from training and one novel object for 5 min. Memory was assessed by measuring object preference using the discrimination ratio (novel object interaction/total object interaction). A mouse was classified as interacting with an object if it approached or sniffed the object. Trials were videotaped (Sony mini-DV camera) and scored off-line.

### Data analysis

Results are expressed as the mean ± SEM. Student's t-test was used to compare pairs of means. In cases with multiple conditions, a two-way ANOVA was used followed by Bonferroni *post hoc* tests when appropriate. A one-way ANOVA was used for single condition analysis followed by Tukey's Multiple Comparison *post hoc* tests when appropriate. Chi-square analysis was used to determine the significance of gene expression changes between WT and DKO mice across time. For novel object recognition, a one sample t-test was run to assess whether percent interaction with an object was different from chance (50%). A p-value of ≤0.05 was considered statistically significant. All statistical comparisons were done with Prism 4 software (GraphPad) unless otherwise stated.

## Results

DKO mice show consistent long-term, but not short-term, CF memory deficits [5]. Impaired gene expression changes within the hippocampus have been correlated with impairments in long-term memory consolidation [30]. Therefore, to assess whether learning deficits resulting from a lack of Ca2+-stimulated AC activity are imparted by impaired gene expression, we obtained amygdala and hippocampal tissue samples from WT and DKO mice at different time points after CF learning. Samples were extracted at baseline (−5 min before training), 0 hr, 1 hr, or 48 hr after a 5 min CF training trial, or at 1 wk after a 5 min CF testing trial, and subjected to microarray analysis and validated with RT-PCR (Fig. S1). The different time points reflect when the different stages of memory processing are thought to occur [31].

**Figure 1:**
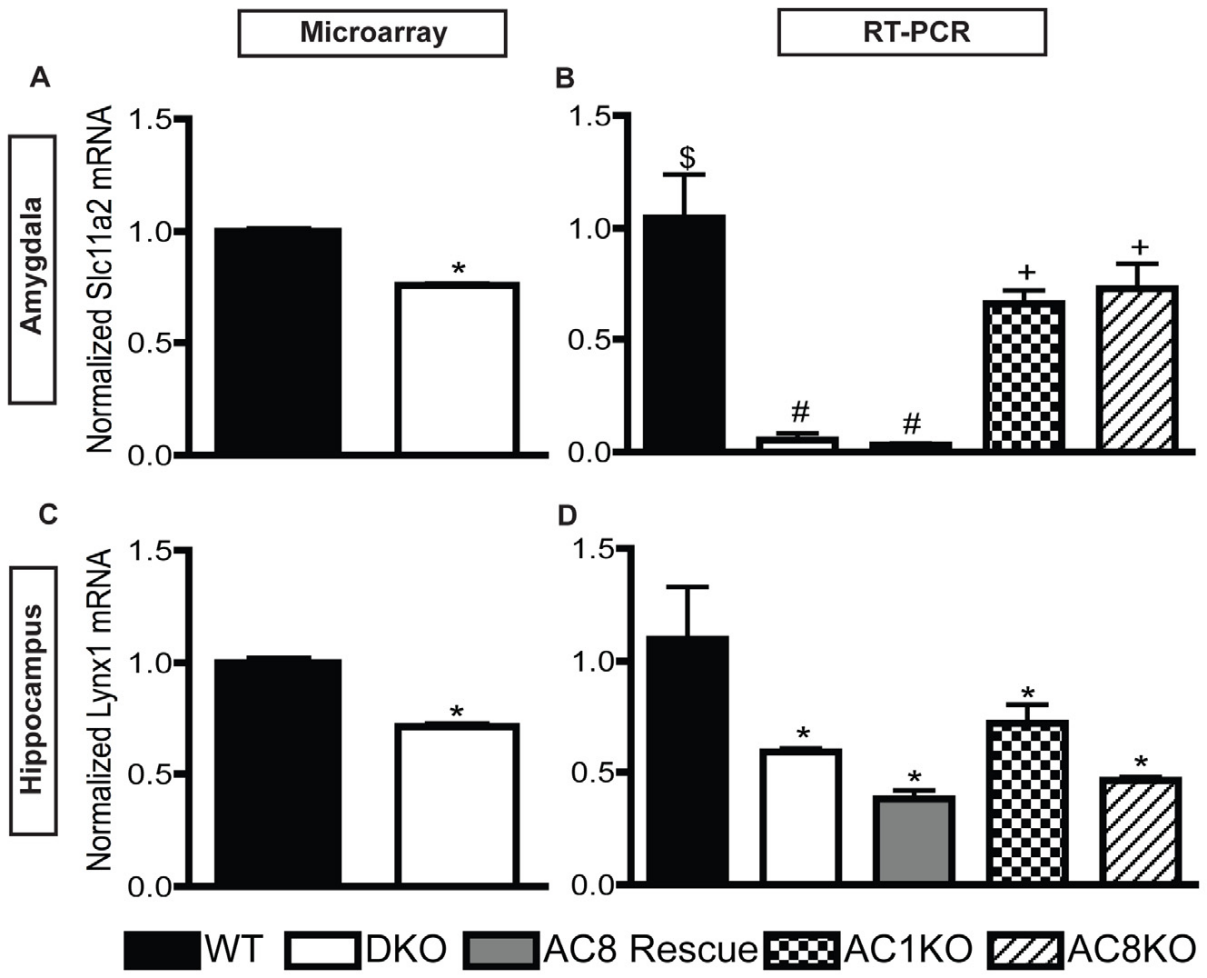
Developmental changes in DKO mice. Two genes identified with microarray analysis and showing the most significant change between WT and DKO mice, (**A**) Slc11a2 (n = 5, *p≤0.0001), with a −1.3 fold change, and (**B**) Lynx1 (n = 5, *p≤0.0001), with −1.4 fold change, were confirmed via RT-PCR. (**C**–**D**) Both RT-PCR results confirmed a significant decrease in Slc11a2 (n = 4–5, $ vs # p≤0.001; $ vs + p≤0.05; # vs +, p≤0.001) and Lynx1 (n = 4–5,*p≤0.05). RT-PCR results also reveal that these gene changes are not rescued in AC8 rescue mice, and therefore, do not modulate the CF learning changes, but rather, probably contribute to baseline changes. No differences in gene expression between AC1KO and AC8KO mice suggest that these genes are not targeted by one specific Ca2+-stimulated AC.

To provide context for gene expression changes found during CF in the DKO mice, we measured basal gene expression differences that were consistent before testing and across all time points after training. As expected, AC8 mRNA was significantly decreased in both the amygdala (−2.76 fc) and hippocampus (−4.06 fc) of DKO mice compared to those of WT mice; however, AC1 only met the FDR threshold in the hippocampus (−2.97 fc), reflecting the low abundance of AC1 in WT amygdala (−1.48 fc, p = 3.6E-04) (Table 1). Decreased expression of a few other genes was observed using microarray in both the amygdala and hippocampus of DKO mice compared to those of WT mice (Table 1; Fig. 1A,C). Confirming our microarray data, we found using RT-PCR that Slc11a2 and Lynx1 are significantly decreased in DKO mice compared to expression levels in WT mice (Fig. 1B, D). Additional RT-PCR analysis of these genes with single knockout animals (AC1KO, AC8KO) suggests that these genes are not targeted by one specific Ca2+-stimulated AC as both single knockouts show similar reduction in expression levels.

### Microarray analysis of gene expression changes after CF learning

#### Amygdala

**Figure 2:**
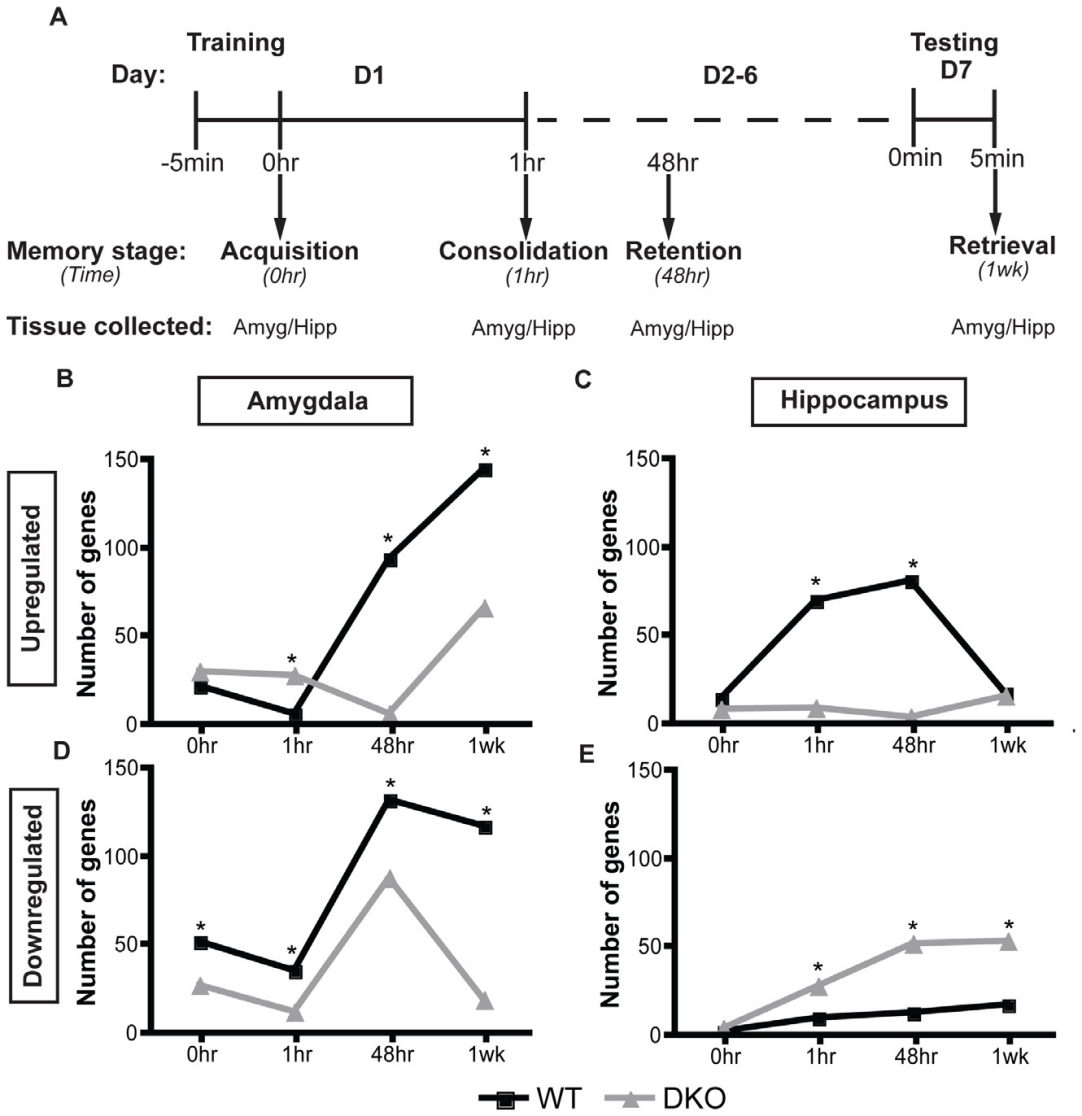
Microarray analysis setup and gene expression changes. (**A**) Amygdala and hippocampal micropunches were taken at baseline and four time points across CF learning (0 hr, 1 hr, 48 hr, 1 wk) in WT and DKO mice (1 array per genotype/brain region/time point). The number of genes showing a ±1.5 fold change from baseline is graphed. (**B**, **D**) WT mice show the largest change in gene expression at the 48 hr and 1 wk time points in the amygdala and (**C**, **E**) at the 1 hr and 48 hr time points in the hippocampus. Overall, DKO mice show a suppression in gene expression changes, except at 1 hr in the amygdala when DKO mice show more gene changes that WT mice (* Chi-square, WT vs DKO p≤0.05).

We find that the aggregate pattern of gene expression changes in the amygdala occurring after CF learning is similar between WT and DKO mice, but the relative number of genes differs between the two genotypes. Chi-square analysis reveals that WT mice have significantly more upregulated genes than DKO mice at 48 hr (94 vs 6; p≤0.05) and 1 wk (145 vs 66; p≤0.05) (Fig. 2B), while DKO mice have more upregulated genes at 1 hr than WT mice (28 vs 6; p≤0.05). Additionally, WT mice have significantly more downregulated genes than DKO mice at every time point in the amygdala (Fig. 2D).

**Figure 3:**
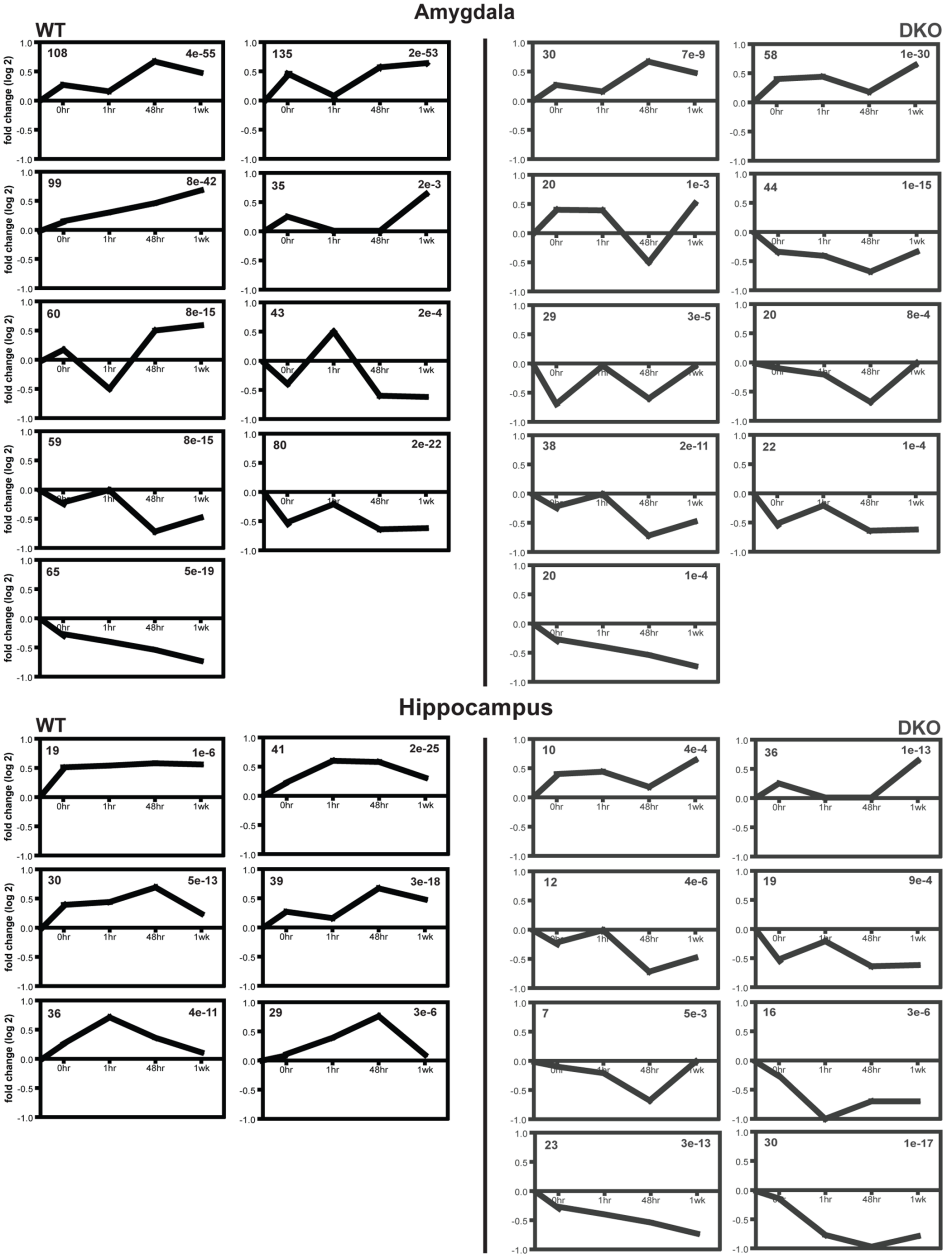
Cluster analysis reveals the most represented expression pattern changes occuring over time after CF learning. Amygdala results reveal a balance between up and downregulated gene changes in WT mice. DKO mice reveal an overall decrease in gene expression within the amygdala over time. Four similar clusters are found between both the WT and DKO mice in the amygdala. WT mice show a consistent upregulation in gene expression over time within the hippocampus; whereas, DKO mice genes tend to be downregulated over time except for two clusters. No two clusters are alike between WT and DKO mice. The number in the upper left corner represents the number of genes in the cluster and the number in the upper right reveals the p-value.

**Figure 4:**
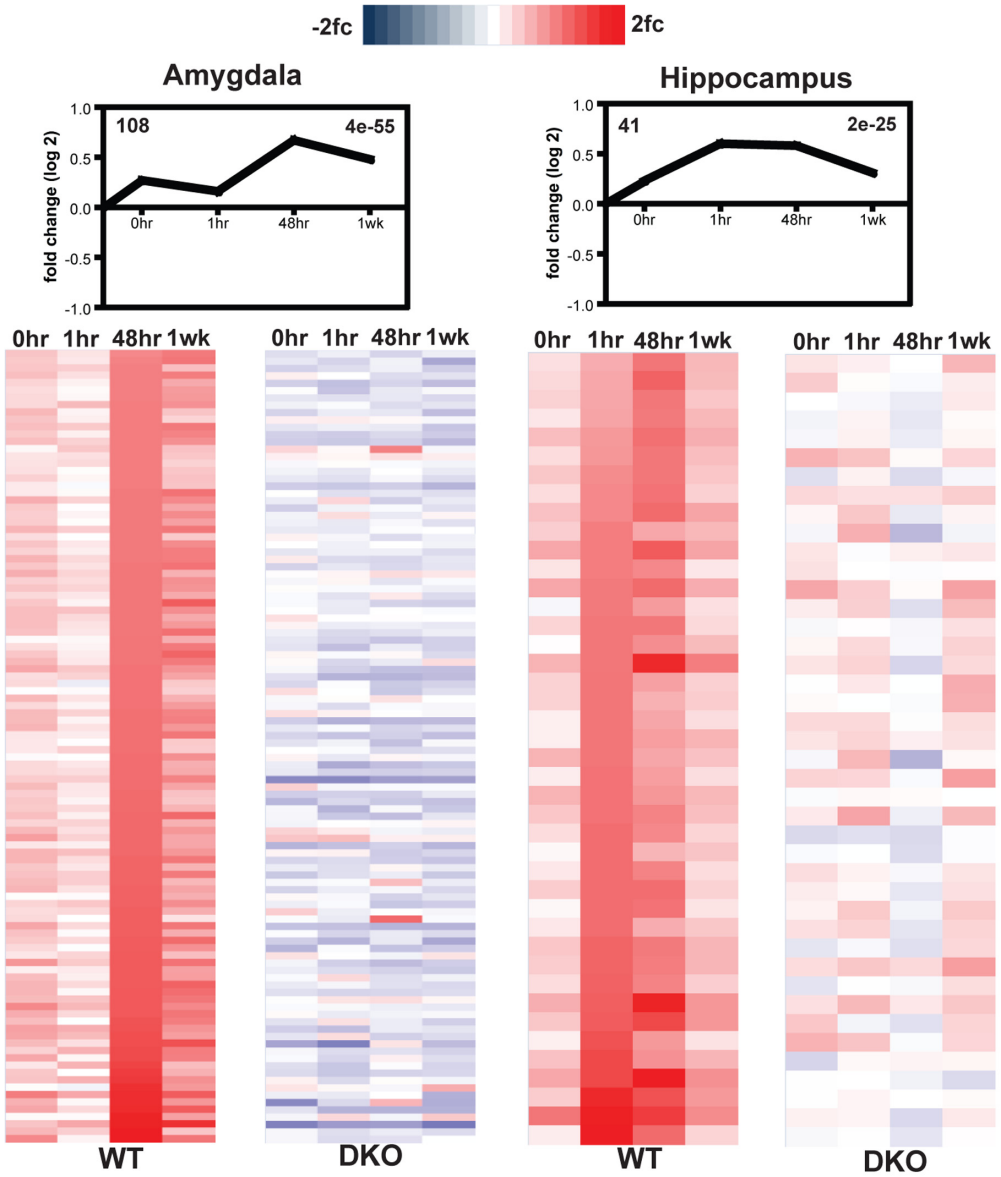
Heat map analysis reveals divergent gene expression changes. A heat map was generated for the most significant cluster in each brain region of WT mice. The gene changes are mapped in the WT mice, and the respective changes in expression of the same genes are mapped in the DKO mice. (**A**) The amygdala results reveal not only a suppression, but opposing regulation in gene expression in DKO mice. (**B**) The hippocampal results reveal an overall suppression in gene changes within DKO mice.

Using the STEM software, we clustered individual genes into groups based on their changes across time and according to whether they showed up or downregulated gene expression (Fig. 3; Tables S8 and S9). Within the amygdala, DKO mice show a predominance of clusters with downregulated expression over time, but 4 out of 9 clusters are similar between WT and DKO mice. Further analysis of these clusters (Table S8) reveals that there is minimal overlap in the specific genes that fall within the same cluster between WT and DKO mice. Therefore, we conducted a heat map analysis on the most significant WT cluster in the amygdala (Fig. 4) to determine how the same set of genes changed between the WT and DKO mice. The WT heat map represents the expression changes of the genes falling into this cluster. The DKO heat map represents expression levels of those same genes and depicts how they are differentially modulated relative to WT mice. Virtually all of these genes show the opposite regulation within the amygdala between WT and DKO mice. This observation is further supported when comparing the ±1.5 fc gene lists between WT and DKO mice within the amygdala (Table S5).

We also determined whether the genes that changed after CF learning in DKO mice were functionally related to the genes that change in WT mice (Table 2). We defined the top 5 functional categories represented by genes changed after CF learning in WT mice and compared the number of genes that changed in DKO mice within the same 5 functional categories. Within the top 5 functional categories, DKO mice show considerably fewer genes relative to WT mice across all time points, except for genes upregulated at 1 hr where DKO mice show a greater number of gene changes, which is consistent with the gene expression pattern changes seen in Figure 2. Functional analysis reveals that the decrease in transcriptional changes may be largely due to the lack of changes in genes that modulate cellular transcription (GO: 0006350). Moreover, DKO mice have deficits in cell signaling as they show a decrease in number of genes that fall into the cell communication (GO: 0007154) and signal transduction (GO: 0007165) functional categories.

**Table 2:** Top 5 functions represented by each respective gene list.

	Number of genes (compared to baseline)							
BIOLOGICAL PROCESS	0 hr		1 hr		48 hr		1 wk	
Amygdala, Upregulated	WT	DKO	WT	DKO	WT	DKO	WT	DKO
Cellular nucleobase, nucleoside, nucleotide and nucleic acid metabolic process (GO:0006139)	5	3	1	11	21	0	34	13
Cell communication (GO:0007154)	2	7	2	6	17	0	34	14
Cell differentiation (GO:0030154)	2	3	2	8	21	0	30	7
Cellular transcription (GO:0006350)	4	2	1	8	19	0	26	7
Signal transduction (GO:0007165)	1	7	1	4	15	0	29	14
**Amygdala, Downregulated**								
Cell communication (GO:0007154)	11	4	5	2	30	18	25	7
Cellular nucleobase, nucleoside, nucleotide and nucleic acid metabolic process (GO:0006139)	4	1	2	1	31	16	28	4
Signal transduction (GO:0007165)	9	4	5	2	27	13	23	7
Protein metabolic process (GO:0019538)	6	4	1	3	26	16	22	4
Transport (GO:0006810)	10	6	7	2	21	16	17	1
**Hippocampus, Upregulated**								
Cellular nucleobase, nucleoside, nucleotide and nucleic acid metabolic process (GO:0006139)	6	0	20	2	21	0	5	4
Cellular transcription (GO:0006350)	5	0	10	2	15	0	5	0
Cell communication (GO:0007154)	3	1	12	1	15	1	4	0
Protein metabolic process (GO:0019538)	2	0	15	2	10	1	2	2
Signal transduction (GO:0007165)	2	1	8	1	13	1	2	0
**Hippocampus, Downregulated**								
Cell communication (GO:0007154)	0	1	3	5	4	12	5	8
Signal transduction (GO:0007165)	0	1	2	5	4	12	5	8
Transport (GO:0006810)	0	0	2	8	3	11	4	9
Cell differentiation (GO:0030154)	0	0	2	3	3	6	2	9

#### Hippocampus

WT mice have significantly more upregulated genes than DKO mice in the hippocampus at 1 hr (70 vs 9; p≤0.05) and 48 hr (81 vs 4; p≤0.05)(Fig. 2C). However, the converse is true for downregulated genes, of which DKO mice have significantly more than WT mice at 1 hr (28 vs 10; p≤0.05), 48 hr (52 vs 13; p≤0.05), and 1 wk (53 vs 17; p≤0.05) (Fig. 2E).

Cluster analysis of individual gene changes in the hippocampus reveals that there are no similar cluster patterns between the WT and DKO mice (Fig. 3; Tables S8 and S9). Only clusters upregulated over time are statistically significant in WT mice with the 1 hr and 48 hr time points showing the largest increases in gene number. DKO mice, however, show a predominance of clusters with downregulated expression over time in the hippocampus. Heat map assessment of the most significant WT cluster in the hippocampus reveals that gene expression changes are attenuated in DKO mice, but the patterns appear in similar directions (Fig. 4). The functional categories of the top clusters in WT hippocampus are similar to those of the amygdala with DKO mice showing a decrease in the number of genes falling within the cellular transcription (GO: 0006350), cell communication (GO: 0007154), and signal transduction (GO: 0007165) categories.

### Normal CF learning displays over-representation of specific transcription factor binding sites in regulated genes

To further elucidate possible mechanisms for the network of gene expression changes associated with CF learning, we assessed the transcription-factor binding sites that were overrepresented in genes either upregulated or downregulated within the amygdala and hippocampus of WT and DKO mice at 48 hr (Top 10 listed in Table 3; full list in Table S10). The DKO mice show no overrepresented transcription factor binding sites in the amydala or hippocampus of upregulated genes at 48 hr; however, this may largely be due to only a small number of genes being upregulated at this time point in DKO mice. Downregulated genes, in contrast, appear to be regulated by a shared group of transcription factors. Nearly all the transcription factor binding sites (9 out of 10) that are overrepresented in the downregulated genes within the hippocampus of DKO mice are found in genes upregulated in the hippocampus of WT mice. This observation is consistent with the shift from predominantly upregulated to downregulated gene expression seen in the DKO mice.

**Table 3:** Top 10 TF binding sites overrepresented in genes regulated at 48 hr.

WT				DKO			
Hippocampus		Amygdala		Hippocampus		Amygdala	
TF binding site	P-value	TF binding site	P-value	TF binding site	P-value	TF binding site	P-value
***Upregulated***							
Pou6f1	2.3E-11	Areb6	7.4E-04	N/A		N/A	
Pou1f1	2.5E-11	Lfa1	1.3E-03				
Tef	4.4E-11	E12	3.5E-03				
Crebatf	4.5E-09	Gata2	3.8E-03				
Gata3	1.9E-08	Hoxa7	5.1E-03				
Dr3	1.5E-07	Pbx1	5.4E-03				
Amef2	1.7E-07	Foxp3	5.8E-03				
Hp1sitefactor	6.0E-07						
Hfh3	7.3E-07						
Atf	1.0E-06						
***Downregulated***							
Pads	1.6E-04	Lhx3	8.9E-10	**TfIIa** *	**1.3E-07**	E47	3.6E-05
Meis1	2.5E-04	Cart1	2.4E-08	**Foxo1** *	**9.4E-07**	**Areb6**	**8.2E-04**
TfIIa	3.8E-04	Pou6f1	3.4E-08	**Gata3**	**1.9E-05**	Cmyb	2.3E-03
Hp1sitefactor	1.3E-03	Sox5	9.6E-08	**Foxo4** *	**3.9E-05**	Crebp1cjun	5.0E-03
Gata1	2.7E-03	Sox9	5.2E-07	Lef1tcf1	4.8E-05	Nmyc	5.1E-03
Areb6	3.1E-03	Hif1	6.7E-07	**Dr3**	**9.3E-05**		
		Nkx61	1.0E-06	**Hp1sitefactor**	**1.6E-04**		
		Hp1sitefactor	2.2E-06	**Foxp3** *	**2.8E-04**		
		Vbp	2.8E-06	**Hfh4** *	**4.3E-04**		
		Gata3	2.9E-06	**Lhx3** *	**7.2E-04**		

The 2.5kbp region upstream region of genes modulated during CF learning was assessed for common transcription factor (TF) binding sites. The significance value takes into consideration the total number of sites in the genes analyzed versus the total number of sites in the whole genome. **Bold** genes represent TF binding sites in DKO mice that are significantly upregulated within each respective WT brain region, while.

*indicates TF binding sites that are significantly upregulated in WT mice, but do not make the top ten list.

### Generation of AC8 rescue mice

**Figure 5:**
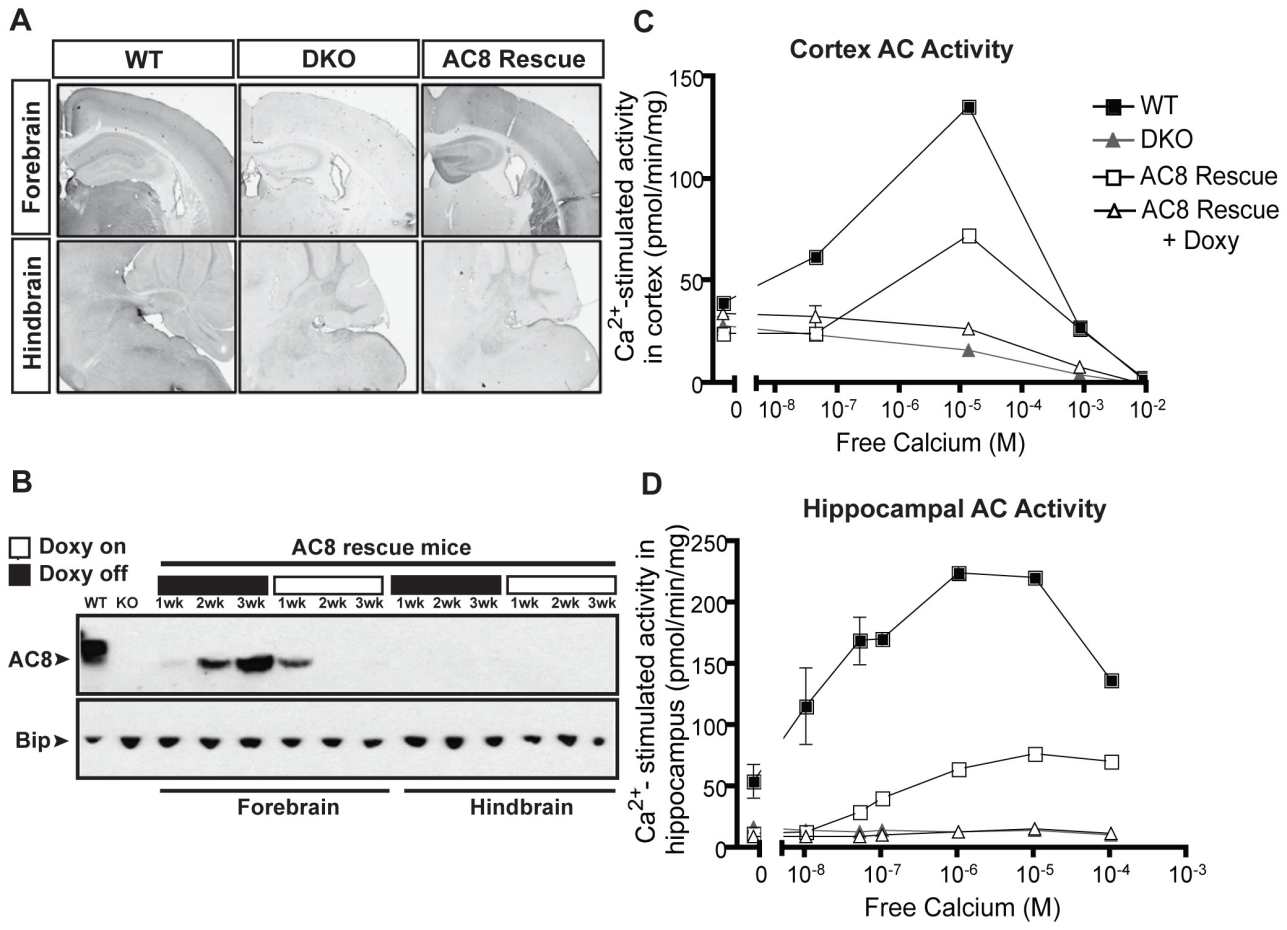
Generation of inducible forebrain-specific AC8 mice on a DKO background. (**A**) Immunohistochemistry results confirm complete absence of AC8 protein levels in DKO mice, while AC8 rescue mice have AC8 replaced within forebrain-specific regions but not in the hindbrain or thalamus. (**B**) Using doxycycline to manipulate AC8 expression within the forebrain, we observed AC8 expression after 2 wk off doxycycline. In contrast, expression begins to rapidly turn off within one week off doxycycline treatment and is undetectable by 2 wk. Hindbrain results reveal no AC8 expression with or without doxycycline treatment. Bip is used as a loading control. The adenylyl cyclase assays in the (**C**) cortex (n = 3/genotype) and (**D**) hippocampus (n = 3/genotype) reveal no Ca2+-stimulated activity in DKO mice or AC8 rescue mice on doxycycline, while activity is rescued to approximately 50% (**C**) and 30% (**D**) of WT levels in AC8 rescue mice off doxycycline.

To functionally test the temporal role of AC activity during CF learning, we restored Ca2+-stimulated AC function at different time points during behavioral testing with a transgenic model of AC8 expression. We generated a system where AC8 is expressed in the forebrain of mice on a DKO background (AC8 rescue mice). We used a doxycycline-regulated system (e.g. tetracycline-off system) to restore AC8 expression in a temporally regulated fashion. Fig. 5A shows the protein distribution of AC8 within WT and DKO mice. Consistent with previous reports, AC8 is localized to the cortex, thalamus, hippocampus, and cerebellum [18]. AC8 rescue mice display AC8 expression within forebrain specific regions, but not within the thalamus or hindbrain. We find that AC8 expression begins to turn on two weeks after taking mice off doxycycline, and conversely, AC8 expression is turned off completely after 2 wk on doxycycline (Fig. 5B). We measured Ca2+-stimulated AC activity in the cortex (Fig. 5C) and hippocampus (Fig. 5D) to determine the magnitude of physiological replacement in AC8 rescue mice. Although expression of AC8 protein in the AC8 rescue mice appears higher than what is present endogenously within the WT mice (Fig. 5A), we found that that overall Ca2+-stimulated AC activity was recovered to approximately 50% and 30% of WT levels in the cortex (Fig. 5C) and hippocampus (Fig. 5D), respectively. As shown previously [5], DKO mice have no measurable Ca2+-stimulated AC activity. Importantly, AC8 rescue mice on doxycycline show no Ca2+-stimulated AC activity, confirming that doxycycline efficiently represses AC8 transgene expression.

### Ca2+-stimulated AC activity is necessary for memory consolidation and retention

**Figure 6:**
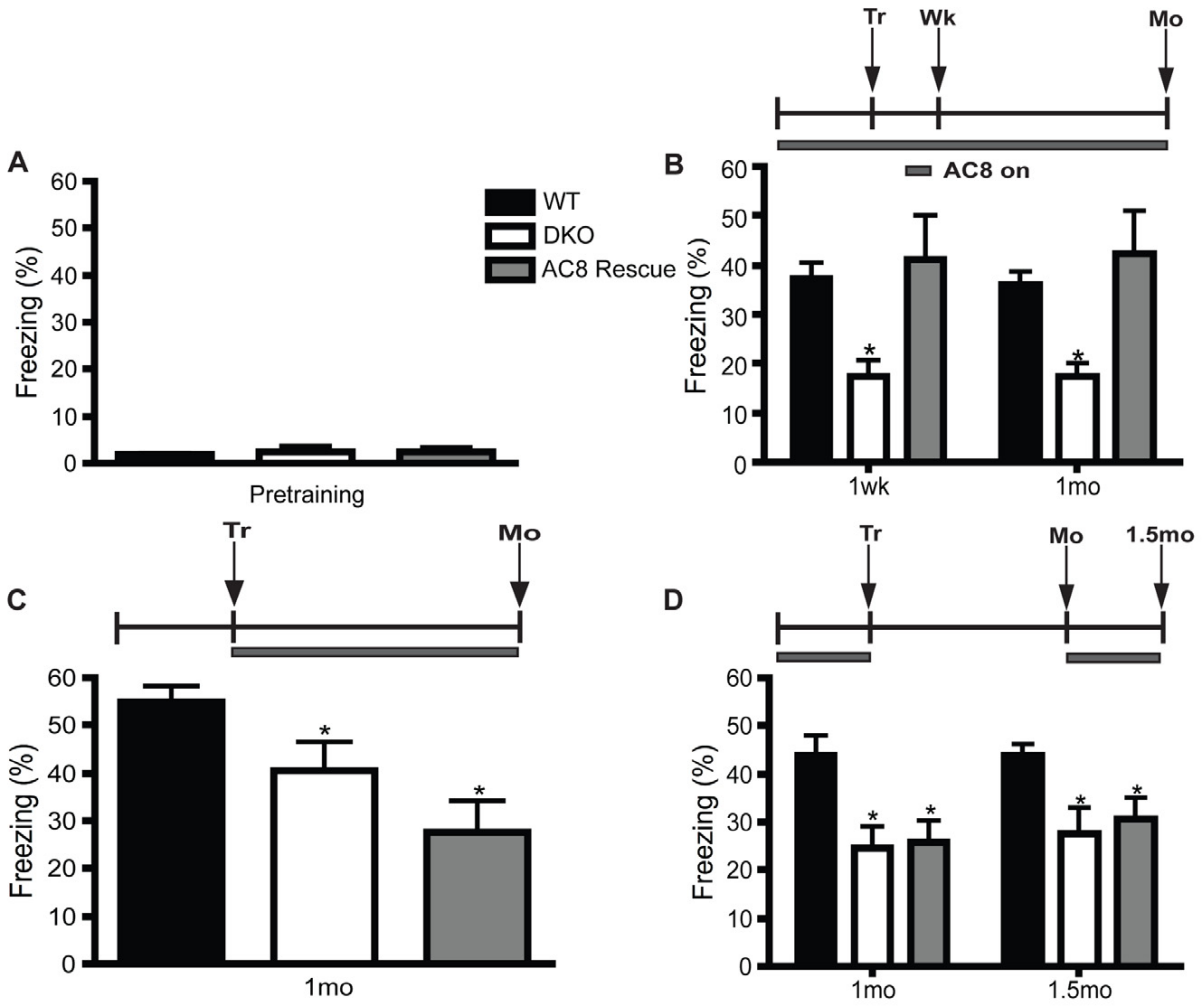
Ca2+-stimulated AC activity is necessary for memory consolidation and retention. Memory changes are assessed by the overall freezing percentage. (**A**) There are no baseline freezing changes between the genotypes (no differences in baseline freezing levels between graphs B, C, or D, so subjects were combined; p>0.05). (**B**) DKO mice show memory deficits at 1 wk and 1 mo compared to WT mice. AC8 rescue mice reveal that replacing AC8 expression within the forebrain of DKO mice throughout training, retention and testing is sufficient to rescue CF memory deficits (n = 9–11/genotype, *p≤0.05 AC8 vs DKO). Turning AC8 off during (**C**) CF training (n = 8–10, *p≤0.05, AC8 and DKO vs WT) or (**D**) during the retention period (n = 9–11, *p≤0.01, AC8 and DKO vs WT) prevents restoration of the memory deficits. Furthermore, AC8 rescue mice still show memory deficits after turning AC8 back on for 2 wks after having it off since training (n = 9–11, *p≤0.05, AC8 and DKO vs WT). AC8 on  =  doxycycline off; Symbols: Tr, training; Wk, week; Mo, month.

After confirming that our AC8 rescue system was able to restore AC8 expression and activity to the forebrain, we turned AC8 activity on and off at different time points throughout CF testing. We found that acutely restoring AC8 continuously during CF training and testing was sufficient to rescue memory deficits seen in DKO mice (Fig. 6B). However, when the AC8 transgene was repressed during training only, memory is impaired (Fig. 6C), suggesting that Ca2+-stimulated AC activity is necessary during memory consolidation. Additionally, when AC8 was kept on during training but turned off during the retention period, memory was again impaired at 1 mo (Fig. 6D).

To investigate if Ca2+-stimulated AC activity was needed just for retrieval, rather than retention, we turned AC8 expression on again and tested mice two weeks later (1.5 mo after training). Suggesting that Ca2+-stimulated AC activity is necessary during memory retention, we found that AC8 expression for two weeks after the 1 mo testing session was unable to rescue CF memory at 1.5 mo (Fig. 6D).

Interestingly, expression of genes identified as consistently developmentally reduced in expression between WT and DKO mice, Slc11a2 and Lynx1, was similar between AC8 rescue and DKO mice, suggesting that these gene changes are not necessary for intact CF memory as expression levels are not rescued in the AC8 rescue mice while learning deficits are rescued (Fig. 1).

**Figure 7:**
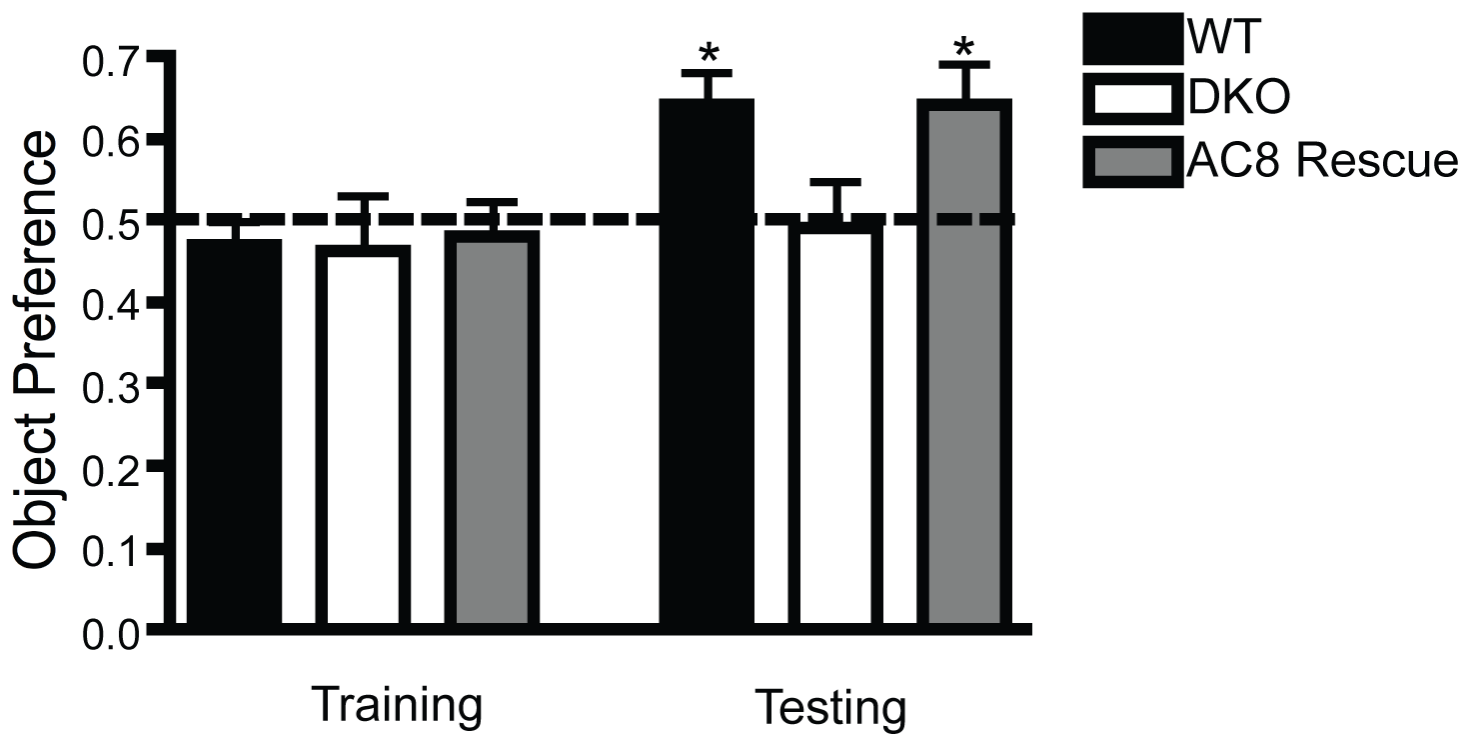
Ca2+-stimulated AC activity is necessary for the acquisition of novel object recognition. The hash mark labels the 50% mark, which represents no preference for an object (object preference  =  novel object interaction/total object interaction). DKO mice show no preference for the novel object when tested 90 min after training (n = 8, object preference is not significantly different than 50%, p>0.05); however, replacing AC8 in the forebrain of DKO mice is sufficient to maintain memory (n = 10/genotype, *1-sample t-test object preference is greater than 50%, p≤0.05).

To evaluate the role of Ca2+-stimulated AC activity during short-term memory, we restored AC8 expression during novel object recognition and compared our results with DKO and WT mice. Consistent with previous reports [32], DKO mice show no bias for the novel object during the testing trial (90 min after training) (Fig. 7). Restoration of AC8 expression in the forebrain of DKO mice elicits a response comparable to WT mice.

To evaluate possible variables that could confound the learning results, we measured baseline freezing in the CF paradigm. These measures were similar between all three groups (Fig. 6A). Additionally, we evaluated anxiety and locomotion levels in open-field testing (data not shown). Indices of anxiety-like behavior, such as time spent in the center vs periphery of the open field, were similar between all three groups, WT (65% periphery vs 35% center ±9.0), DKO (75% periphery vs 25% center ±2.8) and AC8 rescue (75% periphery vs 25% center ±4.5) mice. Locomotion measures were increased (p = 0.02), as assessed by the total number of grid crossings in 5 min, in both the DKO (165 grid crossings) and AC8 rescue mice (166 grid crossings) relative to WT mice (83 grid crossings). However, since AC8 rescue and DKO mice show different learning phenotypes, it is unlikely that the increased locomotion alters interpretation of the memory findings. Basal pain sensation has also been previously studied with no major differences seen between WT and DKO mice [33].

## Discussion

Here, we report that disruption of both Ca2+-stimulated ACs (AC1 and AC8) causes an overall decrease in long-term transcriptional changes after CF learning. While changes between WT and DKO mice are evident at all time points, the most robust disparity occurs at the 1 hr and 48 hr time points, periods when memory consolidation and retention are thought to occur.

Initially, we assessed changes in baseline gene expression to eliminate the possibility that baseline gene expression differences are contributing to the CF deficits in DKO mice. The most notable changes are a decrease in Slc11a2 in the amygdala and Lynx1 in the hippocampus of DKO mice. Both of these proteins may play a role in learning and memory. Slc11a2, a divalent metal ion transporter, causes memory impairments on the Morris water task when disrupted within the forebrain [34], and Lynx1, which enhances nicotinic acetylcholine receptor function, causes memory enhancements on CF when globally disrupted [35]. However, our data show that expression of these genes in AC8 rescue mice is not recovered to WT levels, and since AC8 rescue mice have intact memory, this suggests that these proteins do not cause the CF learning deficits seen in DKO mice.

Past literature supports the theory that Ca2+-stimulated AC activity plays a pertinent role in learning and memory. Although disrupting just one of the two Ca2+-stimulated ACs can cause memory impairments [6], data show that AC single knockout mice show intact CF behavior while DKO mice show alterations in CF behavior [5]. Testing in the CF paradigm reveals that DKO mice have intact memory at 24 hr, but impaired memory at 1 wk. Moreover, when tested on a novel object recognition paradigm, DKO mice have impaired object recognition at 1 hr, but not 5 min [32]. Overall, these data suggest that Ca2+-stimulated AC activity is not necessary for acquisition of a task, but necessary for long-term consolidation of a memory and that a single AC (AC1 or AC8) can compensate for loss of the other AC. Our data support these findings as AC8 rescue mice that have AC8 turned off during the consolidation CF training period show memory impairment, while AC8 transgene expression throughout training and testing restores long-term conditioned responses. Additionally, we recapitulate the novel object recognition data with DKO mice showing impairments, but also provide evidence that memory can be restored if AC8 expression is turned on in the forebrain before training. Overall, our learning and memory data suggests an impairment at the consolidation phase.

The observation that Ca2+-stimulated AC activity is important for learning and memory is supported by the microarray data. Transcriptional changes within hours after exposure to a learning paradigm is required for the consolidation of long-term memory [14], [15], [16]. Moreover, recent evidence shows that an age-related decline in memory is correlated with decreased hippocampal transcriptional changes [30]. Together, these data suggest that the lack of long-term transcriptional changes displayed by DKO mice throughout the CF learning process contribute to the learning deficits seen at 1 wk post CF training. Furthermore, the residual transcriptional changes that do occur in DKO mice are mainly suppression of expression. This is evident in Figure 2 as upregulated gene expression is overall dampened over time in both the amygdala and hippocampus of DKO mice.

DKO mice display an impairment in transcriptional changes at 1 hr, a period when memory consolidation is occurring; therefore, the overall suppression in transcriptional changes may be contributing to the inability of these mice to form a strong enough memory in order to retain it for longer than 24 hr. DKO mice show an increased number of genes changing relative to WT mice at 1 hr in the amygdala. This data suggests that the amygdala in DKO mice may be sufficiently activated to form a stress response to the aversive shock administered during CF training. Therefore, the activation of the amygdala may be sufficient to retain the CF memory for short periods, such as seen at 24 hr [5], but the overall lack of hippocampal activation may lead to impairments in the overall strength of the memory formed.

Not only is memory consolidation impaired in DKO mice, but memory retention is as well. This is evident when Ca2+-stimulated activity is turned off in AC8 rescue mice after CF training. These mice fail to show a normal conditioned response when tested 1 wk after training. These data are in apparent conflict with analysis of passive avoidance behavior in DKO mice. DKO mice exhibit impaired passive avoidance behavior that can be restored when forskolin is administered one time into the hippocampus of DKO mice 15 min prior to training, even when memory testing occurs as long as 8 days after training [5]. This would imply that Ca2+-stimulated AC activity is only necessary for consolidation of a memory as the memory was retained 8 days after initial cAMP activation. However, passive avoidance is a less complex task than CF learning; therefore, a one time injection of forskolin may be sufficient to rescue memory deficits in passive avoidance, but for CF learning, a more sustained activation of biological pathways might be necessary to maintain the memory. The present data suggest that a maintenance of positive gene regulation is necessary as DKO mice show an overall lack of upregulated gene changes, but downregulated gene changes are maintained in the hippocampus. This is further seen by the overall lack of common transcription factor binding sites found in the genes upregulated within the amygdala or hippocampus of DKO mice at 48 hr. Moreover, many of the transcription factors that modulate positive gene expression are found overrepresented in the genes that modulate negative gene expression within the hippocampus of DKO mice. Finally, the gene cluster analysis in DKO mice reveals that gene expression is decreased in most of the significant clusters at 48 hr, and functionally, there is at most only 1 gene found within the top 5 functions represented by genes modulated in WT mice. The lack of transcriptional changes at the period when memory is supposed to be maintained supports the behavioral observations that Ca2+-stimulated AC activity is needed for memory retention.

Finally, the present functional analysis data suggests that the lack of transcriptional changes may be contributing to deficits in communication occurring at the level of the synapse. WT mice show a large number of transcriptional changes that functionally contribute to cell communication and signal transduction; whereas, DKO mice show a minimal number of transcriptional changes related to these functions.

In this study, we demonstrate that Ca2+-stimulated AC activity is necessary for memory consolidation and retention during CF learning as well as the long-term transcriptional changes that are associated with CF memory processing. Deletion of both AC1 and AC8 leads to deficits that result in an inability to maintain the CF memory, but this is rescued when AC8 is replaced within the forebrain of mice on a DKO background. Therefore, Ca2+-stimulated AC activity in the forebrain is sufficient for normal CF learning, and the role is acute rather than resulting from developmental deficits (in the DKO mice). The network of changes that we define should serve as a valuable resource for studies of learning and memory in other genetically altered systems allowing fundamental common mechanisms to emerge.

## Supporting Information

Figure S1 Microarray values correlate with the RT-PCR values, validating the microarray results. A random selection of genes was evaluated in WT and DKO mice across all time points in the amygdala and hippocampus to confirm that microarray results correlated with RT-PCR results across all arrays. The graph represents the microarray raw intensity values on the x-axis and the CT value of each experimental primer minus the CT value of the control primer, GAPDH, on the y-axis. An inverse relationship (R2 = 0.78) confirms results as microarray value intensity should increase when CT value decreases (a lower CT value corresponds with increased mRNA).
(0.13 MB TIF)

Table S1 The fold change in the amygdala of WT mice for each respective probe. The table lists all the probes with the respective fold changes at each time point relative to baseline.
(4.58 MB XLS)

Table S2 The fold change in the amygdala of DKO mice for each respective probe. The table lists all the probes with the respective fold changes at each time point relative to baseline.
(4.60 MB XLS)

Table S3 The fold change in the hippocampus of WT mice for each respective probe. The table lists all the probes with the respective fold changes at each time point relative to baseline.
(4.60 MB XLS)

Table S4 The fold change in the hippocampus of DKO mice for each respective probe. The table lists all the probes with the respective fold changes at each time point relative to baseline.
(4.60 MB XLS)

Table S5 ±1.5 fold change gene list for the amygdala. The table lists all the genes in the amygdala that show at least a ±1.5 fold change relative to baseline at each time point after CF learning.
(0.14 MB XLS)

Table S6 ±1.5 fold change gene list for the hippocampus. The table lists all the genes in the hippocampus that show at least a ±1.5 fold change relative to baseline at each time point after CF learning.
(0.06 MB XLSX)

Table S7 The primer sequences used for RT-PCR analysis.
(0.02 MB XLS)

Table S8 Amygdala gene cluster list. The table lists the genes represented within each cluster in the amygdala.
(0.26 MB XLS)

Table S9 Hippocampus gene cluster list. The table lists the genes represented within each cluster in the hippocampus.
(0.16 MB XLS)

Table S10 All the significant transcription factor binding sites found overrepresented in genes regulated at 48 hr.
(0.05 MB XLSX)
