# Supplementary figures and images for: Temporal and Regional Regulation of Gene Expression by Calcium-Stimulated Adenylyl Cyclase Activity during Fear Memory
Source: PLoS One. 2010 Oct 14;5(10):e13385. doi: 10.1371/journal.pone.0013385 (PMC2954788; doi:10.1371/journal.pone.0013385)

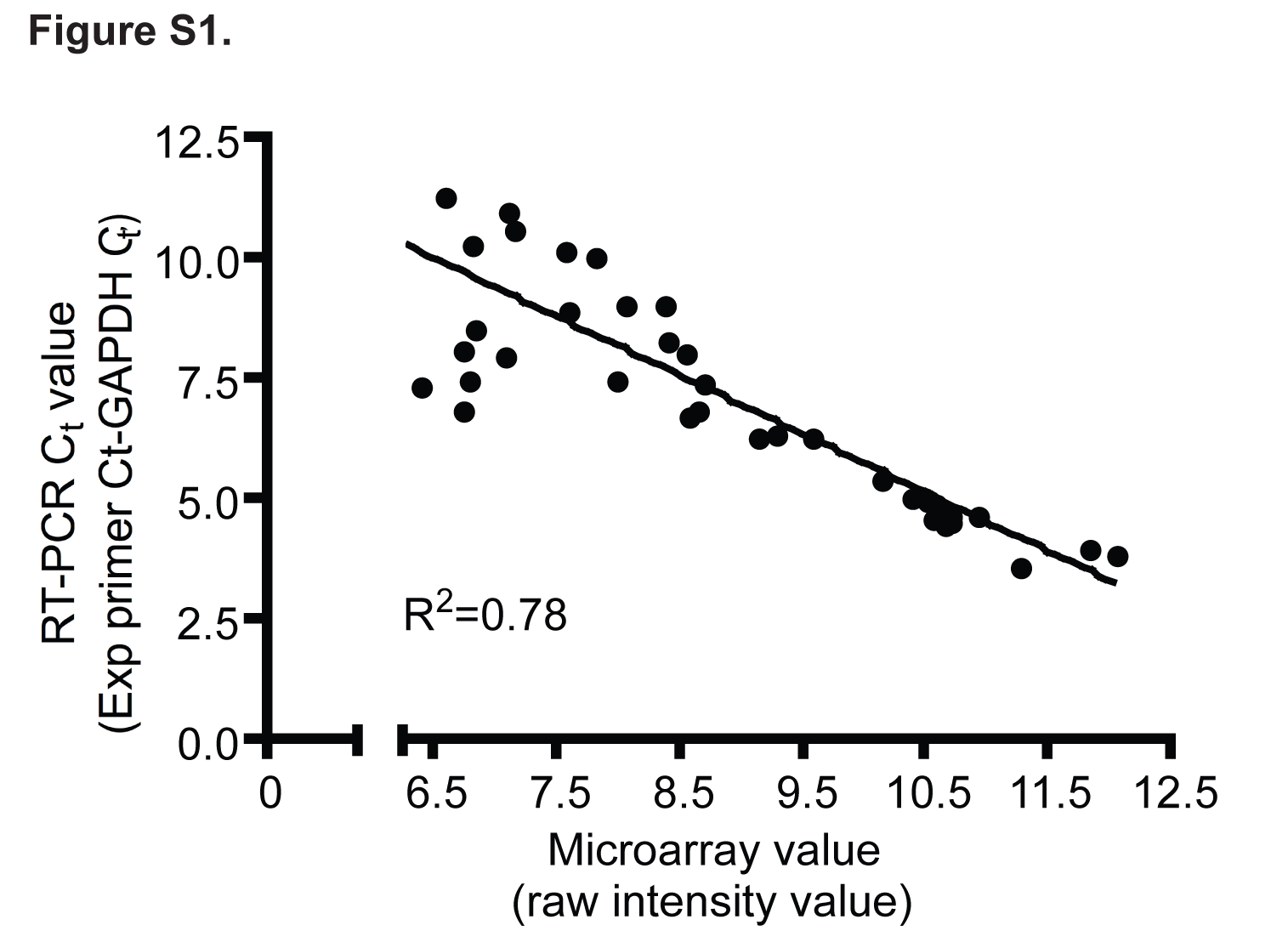

Supplement: Figure S1 — Microarray values correlate with the RT-PCR values, validating the microarray results. A random selection of genes was evaluated in WT and DKO mice across all time points in the amygdala and hippocampus to confirm that microarray results correlated with RT-PCR results across all arrays. The graph represents the microarray raw intensity values on the x-axis and the CT value of each experimental primer minus the CT value of the control primer, GAPDH, on the y-axis. An inverse relationship (R2 = 0.78) confirms results as microarray value intensity should increase when CT value decreases (a lower CT value corresponds with increased mRNA). (0.13 MB TIF) [file pone.0013385.s001.tif]
